# Correlation effects and many-body interactions in water clusters

**DOI:** 10.3762/bjoc.14.83

**Published:** 2018-05-02

**Authors:** Andreas Heßelmann

**Affiliations:** 1 Lehrstuhl für Theoretische Chemie, Universität Erlangen-Nürnberg, Egerlandstr. 3, 91058 Erlangen, Germany

**Keywords:** dispersion, many-body effects, water

## Abstract

**Background:** The quantum-chemical description of the interactions in water clusters is an essential basis for deriving accurate and physically sound models of the interaction potential for water to be used in molecular simulations. In particular, the role of many-body interactions beyond the two-body interactions, which are often not explicitly taken into account by empirical force fields, can be accurately described by quantum chemistry methods on an adequate level, e.g., random-phase approximation electron correlation methods. The relative magnitudes of the different interaction energy contributions obtained by accurate ab initio calculations can therefore provide useful insights that can be exploited to develop enhanced force field methods.

**Results:** In line with earlier theoretical studies of the interactions in water clusters, it has been found that the main contribution to the many-body interactions in clusters with a size of up to *N* = 13 molecules are higher-order polarisation interaction terms. Compared to this, many-body dispersion interactions are practically negligible for all studied sytems. The two-body dispersion interaction, however, plays a significant role in the formation of the structures of the water clusters and their stability, since it leads to a distinct compression of the cluster sizes compared to the structures optimized on an uncorrelated level. Overall, the many-body interactions amount to about 13% of the total interaction energy, irrespective of the cluster size. The electron correlation contribution to these, however, amounts to only about 30% to the total many-body interactions for the largest clusters studied and is repulsive for all structures considered in this work.

**Conclusion:** While this shows that three- and higher-body interactions can not be neglected in the description of water complexes, the electron correlation contributions to these are much smaller in comparison to the two-body electron correlation effects. Efficient quantum chemistry approaches for describing intermolecular interactions between water molecules may therefore describe higher-body interactions on an uncorrelated Hartree–Fock level without a serious loss in accuracy.

## Introduction

The description of the intermolecular interactions between water molecules is essential for an understanding of the structures and properties of water through the different stages of assemblies, from the dimer over the liquid phase to the bulk phase. Moreover, many chemical processes are explicitly or implicitly influenced by a water environment. An example for this is the hydrogen-bond cooperativity effect that can have a significant impact on the properties of the bare solute molecules [1]. In order to describe such phenomena, computer simulations have become an indispensable tool, since they enable a description of water on a molecular level that often can provide further insights than are accessible from spectroscopic measurements.

The basis for such simulations are the so-called force fields that describe both the covalent as well as the noncovalent interactions within the system. These commonly depend on a number of empirical parameters that are determined either by a fit to experimentally known liquid or bulk properties, or by fitting to energies from ab initio quantum chemistry methods. The most popular potentials for water are the TIP3P [2], TIP4P [2–3] and TIP5P [4–5] force fields, which are based on a modeling of the water pair potential using an electrostatic contribution described by interacting point charges and a van der Waals interaction contribution using Lennard-Jones potentials. In more advanced ab initio water pair potentials the force field is fitted to high-level quantum chemistry results for the water dimer. Force fields belonging to this category are, e.g., the TTM3-F and TTM4-F models [6–7], the AMOEBA force field [8–9], the DPP2 model [10] and various force fields derived by Szalewicz and co-workers [11–13]. There also exist a number of pair potentials that go beyond the point charge approximation [14].

A comparison with high-level coupled-cluster energies for a large number of water dimers and tetramers has revealed, however, that polarisation effects, which are not accounted for in the classical point charge potentials, are essential to describe the structures of (H_2_O)_2_ and (H_2_O)_3_ in many different conformations [15]. A rather good correlation between the coupled-cluster energies and the force-field energies is found for the polarisable AMOEBA2003 [9,16] and TTM4-F [17] potentials both for the dimer and for the trimer. Both these methods are based on the induced dipole scheme in which polarisable point dipoles, which are assigned to the molecules, interact with the surrounding electric field and are computed in a self-consistent manner. While these force fields, too, rely on a certain degree of empiricism, a number of other force field exist that aim at a more physically sound decomposition of the interaction energy into distinct contributions. Examples for such force fields are the sum of interaction between fragments (SIBFA) [18–20] and the effective fragment potential (EFP) [21] method. The most recent version of the latter, EFP2, can describe both (long-range) polarisation as well as charge-transfer interactions. The latter was found to yield a significant contribution to the interaction energy of the water dimer [22].

However, one of the most significant results of [22] was that the dipole–quadrupole polarisability term of the multipole expansion of the dispersion interaction, which is usually neglected in force fields or dispersion corrected DFT methods, is a quite large positive (for the clusters considered) and anisotropic contribution to the interaction energy of small water clusters [22]. It was found to be almost half of the magnitude of the leading-order dipole–dipole term. It was therefore concluded by Guidez et al. that this term should not be neglected in the description of the interactions in water.

Another challenge for water models is the description of nonadditive many-body terms to the interaction energy [23–25]. It has been found that these contribute 15% to the total interactions in the condensed phase [26] and this amount even increases to 17–30% for small water clusters [26–28]. Explicit evaluations of three-body interaction energy terms using symmetry-adapted perturbation theory (SAPT) for the water trimer have revealed that the strongest contribution to the three-body energy originates from the polarisation (induction) energy while the three-body dispersion interaction is rather small [28–29]. Moreover, many-body exchange effects, including exchange–induction and exchange–dispersion, are relatively large yet cancel each other due to opposite signs at the uud minimum configuration [29] (“uud” indicates that two “free” hydrogen atoms point above the plane formed by the three oxygen atoms (u→up) and one below it (d→down) [30]). In the study by Hodges et al. it has been shown for several structures of the water tetramer that total four-body interactions are in most cases much smaller or even negligible compared to the three-body interactions [27]. The only exception to this was observed for the squared geometry in which the hydrogen bonds act cooperatively to enhance the induction energy. In a recent work by Hapka et al. it was shown that also standard density functional theory methods are able to describe nonadditive effects to the interaction energy quite well for hydrogen-bonded clusters, yet, fail to do so for dispersion bound complexes [31]. The common conclusion from the quantum chemistry studies of small water clusters was that damped classical polarization models should be able to accurately capture the nonadditive effects for larger clusters of water, because of the fact that short-range contributions, including many-body exchange effects, grow less strongly with the size of the system than induction, dispersion or electrostatic interaction energies [32].

In this work we will study the impact of electron correlation effects and many-body interactions on the structures and energies of water clusters (H_2_O)*_n_* with cluster sizes ranging between *n* = 2 and *n* = 13. In doing so, we focus on the importance of dispersion energy contributions to the interaction energy, including two-body and many-body dispersion effects. It will be shown that overall electron correlation effects have only a minor impact on the orientation of the water molecules in the various minimum structures of the clusters. Dispersion interactions, however, make up a significant contribution of about 60% to the total interaction energies in larger water clusters and lead to a compression of the cluster sizes on average. The total magnitude of the electron correlation contribution to the interaction energy is, however, only about half the size of the sum of the two-, three- and four-body dispersion interactions, i.e., the large dispersion interaction contribution is strongly quenched by further repulsive correlation contributions. Noting that the correlation effect to the molecular dipole moment of the water molecule reduces the dipole moment from the Hartree–Fock method by about 0.13 Debye [33], it can be assessed that a fraction of this repulsive correlation contribution originates from the reduction of the electrostatic interaction energy [34]. Empirical models for water that are based on a fitting to ab initio results therefore have to take the influences of the different correlation effects carefully into account.

## Many-Body Expansion of the Interaction Energy

Consider a cluster system containing *N* molecules. The total interaction energy of this system is given by

[1]
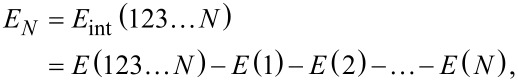


with *E*(123…*N*) denoting the total energy of the system and *E*(*A*) (with *A* = 1,2,…) denoting the one-body clusters (monomers). The idea of the many-body expansion is to decompose the total interaction energy into terms arising from the two-body, three-body, four-body, etc. interactions, so that the total energy is given by

[2]
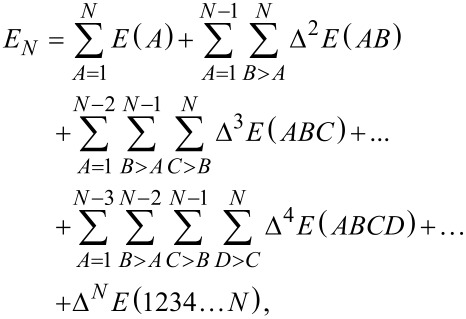


with Δ*^M^* denoting the *M*-th body interaction contribution. These describe the change of the total energy of the system due to the interactions of the *M*-th body clusters. The first three terms are defined as

[3]
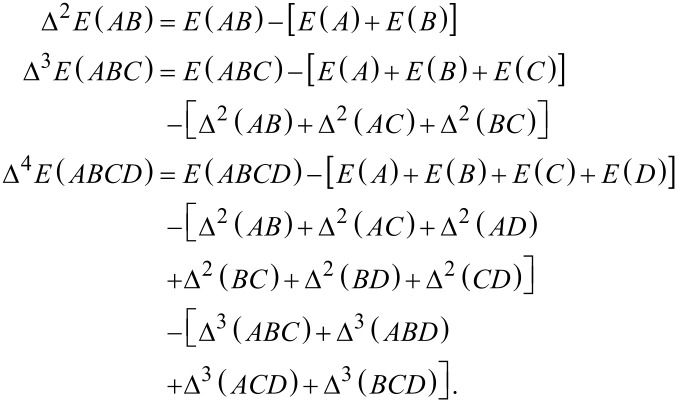


**Figure 1:**
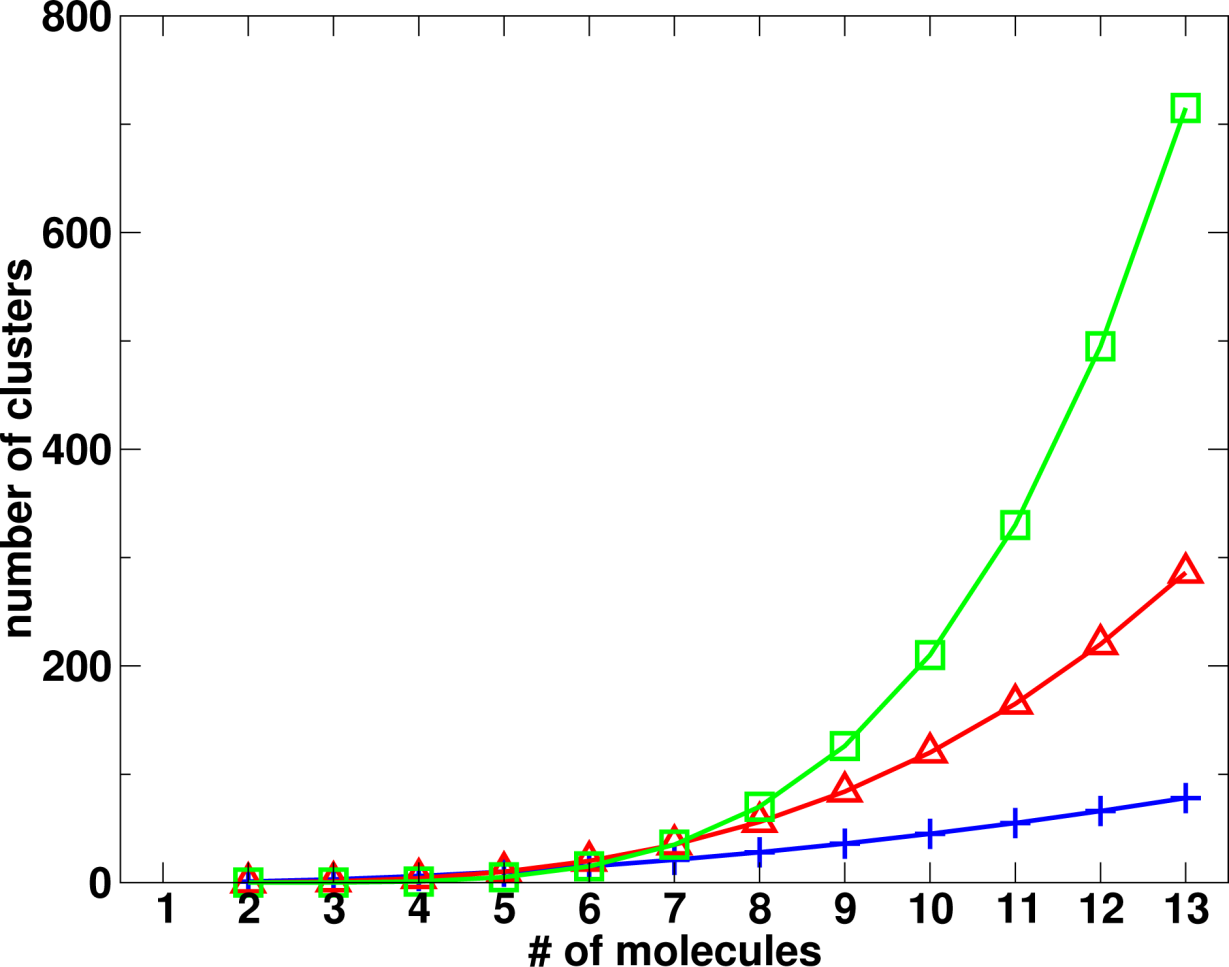
Number of two-body, three-body and four-body clusters for systems with up to 13 molecules.

As shown in Figure 1, the number of three- and four-body clusters increases much more rapidly with the size of the system than the number of two-body clusters. In spite of this, however, a truncation of the expansion of Equation 2 after the two-body term will usually capture 90% and more of the total interaction energy. This originates in part from the fact that for larger many-body systems many three-body and four-body clusters possess structures with far distant molecules. Moreover, the individual interaction energy contributions to the three- and higher-body interaction energy tend to be much smaller than the sum of the two-body interaction energies, as will be shown in this work.

## Method

The calculations in this work have been performed using the EXX-RPA (exact-exchange random-phase approximation) electron correlation method [35–37], which is based on an exact-exchange Kohn–Sham reference determinant [38–43]. The total energy of the EXX-RPA method is given by

[4]
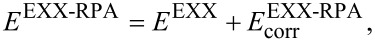


where *E*^EXX^ denotes the energy of the EXX reference determinant and 
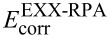
 is the EXX-RPA correlation energy. Like the Hartree–Fock method, the EXX method lacks the description of electron correlation effects, i.e., electron–electron interactions beyond zeroth and first order in the Coulomb interaction. Thus, any inter- and intramolecular correlation effects of the EXX-RPA method are described by the correlation term 
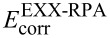
 of Equation 4.

In order to analyze individual interaction energy contributions of the two-body clusters, the DFT-SAPT method [44–52] method has been used. With this method, the interaction energy of a dimer system *AB* is given by

[5]
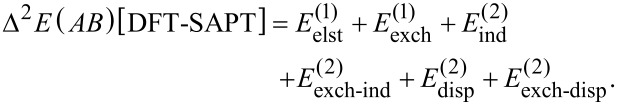


The interaction energy terms in Equation 5 are: 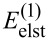 electrostatic interaction energy, 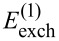 first-order exchange interaction energy, 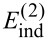 induction energy, 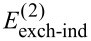 exchange-induction energy, 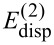 (two-body) dispersion energy and 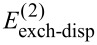 exchange-dispersion energy. The exchange interaction energy terms in Equation 5 are short-range contributions to the interaction energy and stem from a tunneling of the electrons between the two monomers. They quickly decay exponentially with the distance of the interacting systems. The superscripts (1) and (2) denote the order of the individual terms with respect to the interaction energy operator.

In this work all terms in Equation 5 have been computed using EXX monomer wave functions. Moreover, a time-dependent EXX (TDEXX) response approach was used to compute the second-order interaction energy contributions [53–59]. Due to this choice, the subtotal

[6]
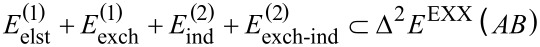


approximates the EXX interaction energy between the monomers *A* and *B*. The difference between Δ^2^*E*^EXX^(*AB*) and these interaction energy terms can be interpreted as higher-order exchange–induction interaction terms not accounted for by the DFT-SAPT method (when truncated at second order).

Moreover, the sum of the dispersion and exchange–dispersion energy

[7]
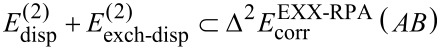


is a fraction of the EXX-RPA correlation energy contribution to the intermolecular interaction energy, since both the DFT-SAPT terms as well as the EXX-RPA correlation energy are computed with the exact-exchange response kernel [53–54]. Here, the main difference of the quantities on the left-hand and right-hand side of Equation 7 stems from the correlation energy contributions to the electrostatic, induction and their exchange interaction energy counterparts. Note that an analogous decomposition of the supermolecular interaction energy into distinct terms is also possible for the second-order Møller-Plesset perturbation theory method [60].

Finally, we have also computed three-body and four-body dispersion interaction energies that contribute to the three-body and four-body interaction energy terms:

[8]
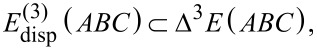


[9]
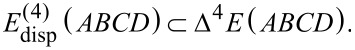


Thus, it will be possible to evaluate the importance of dispersion interactions both for the total many-body interaction energy as well as for its correlation interaction contribution.

## Computational Details

**Figure 2:**
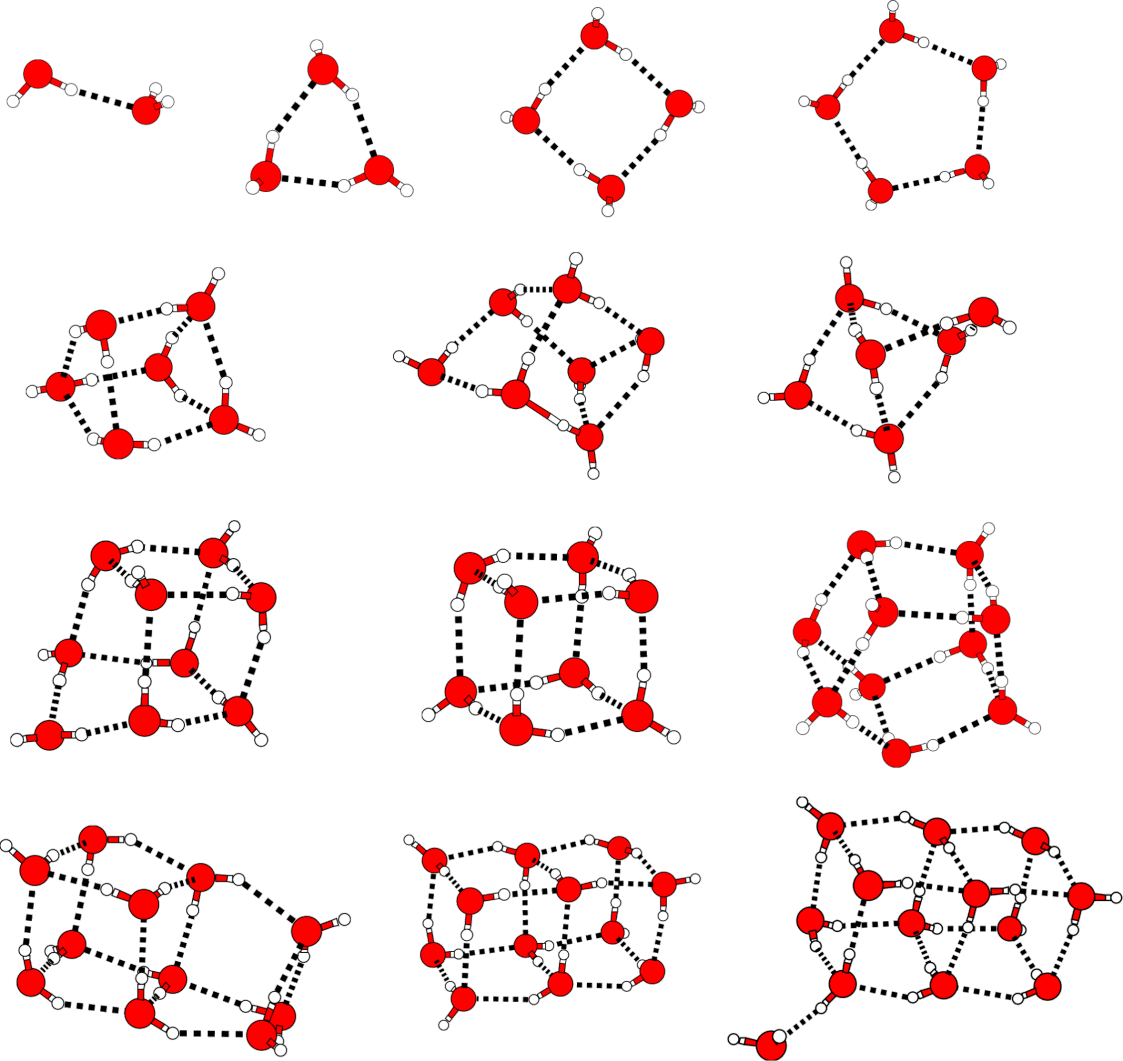
Water clusters from [23] studied in this work.

**Figure 3:**
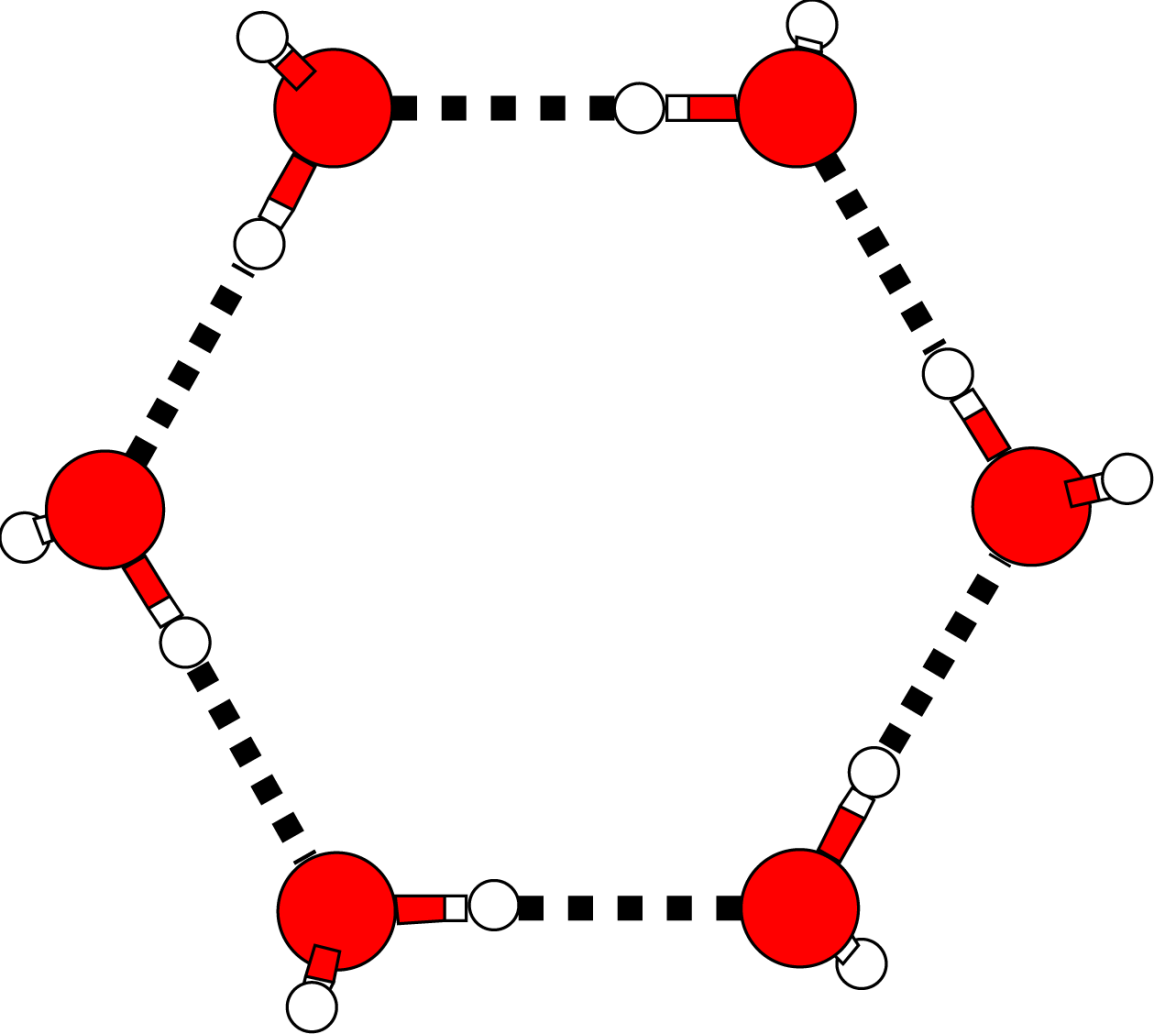
Cyclic-chair structure of the water hexamer.

The structures of the water clusters have been taken from the work of Maheshwary et al., see Figure 2. This set of structures contains both the prism form as well as the cage form of the water hexamer. These are almost isoenergetic and form the first noncyclic global minimum structures of (H_2_O)*_x_*. In addition, the cyclic-chair conformation of the hexamer is considered, too, in this work, since it is known that this structure is characterised by strong many-body interactions [61–63]. For consistency, the cyclic-chair geometry has been optimized on the same level that was used in [23], namely Hartree–Fock employing the 6-31G(d,p) basis set [64]. The resulting structure is shown in Figure 3.

All calculations of this work were performed using the aug-cc-pVTZ basis set by Dunning and co-workers [65]. In order to correct the basis set error of the correlation energy terms, these were extrapolated using a double-ζ to triple-ζ two-point extrapolation using the formula from Bak and co-workers [66]. Core electrons have been kept frozen in these calculations.

The counterpoise correction of Boys and Bernadi [67] has been employed in all sub-cluster calculations to reduce the basis set superposition error. All calculations have been performed using a developers version of the Molpro quantum chemistry package [68–69].

## Results and Discussion

### Influence of correlation effects on the structures

**Figure 4:**
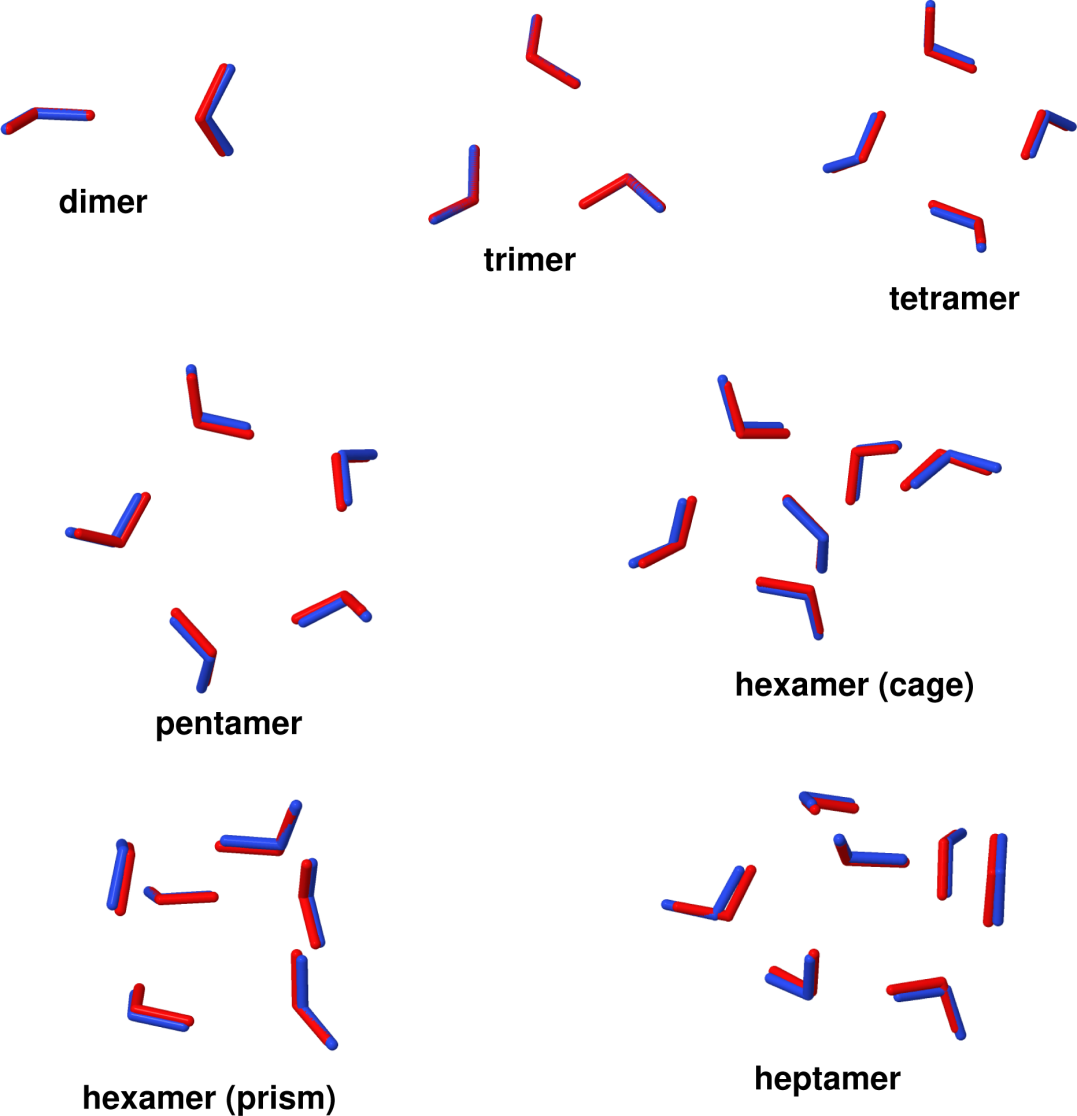
Comparison of the structures of the first seven water clusters optimized on the Hartree–Fock and MP2 levels of theory. Blue: HF, red: MP2 (def2-TZVP basis set).

To investigate the influence of electron correlation effects (including intermolecular dispersion interactions) on the structure of the water clusters, we have reoptimized the structures from [23] on the Hartree–Fock (HF) level using the def2-TZVP basis set [70]. These modified HF geometries were then further optimized with the MP2 (second-order Møller-Plesset perturbation theory) method, which takes electron correlation effects into account at the second order. The resulting structures for the dimer up to the heptamer are shown in Figure 4. Here, for better visibility, the HF structures are colored in blue and the MP2 structures in red. They are superpositioned in such a way that the average distances of the atoms of the respective geometries are minimised.

As can be seen in Figure 4, the electron correlation effects described by the MP2 method hardly change the global structures of the various clusters. This indicates that they mainly originate from electrostatic and induction interactions that can already be described reasonably well with the HF method. However, except for the trimer, where HF and MP2 geometries hardly differ from each other, one can observe that the correlation effects lead to a compression of the structures relative to the ones obtained with HF. This can be attributed to dispersion interactions between the molecules which is an additional attractive interaction energy contribution not accounted for by the HF method, see also below.

**Figure 5:**
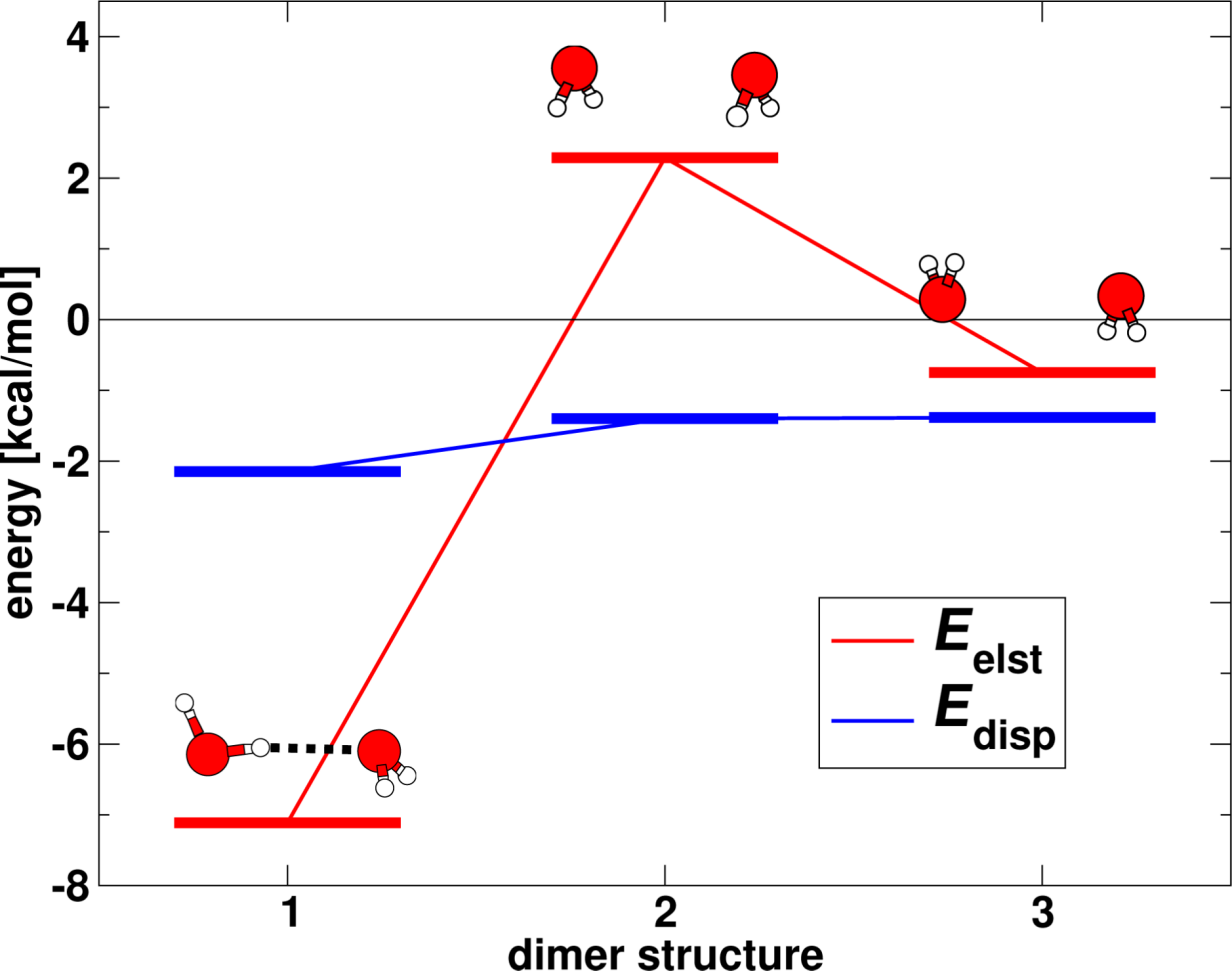
Electrostatic and dispersion interaction energy for three different structures of the water dimer. In all structures the oxygen–oxygen distance is equal to 2.98 Å. The first structure corresponds to the equilibrium (aug-cc-pVTZ basis set).

The dependence of the electrostatic and dispersion energy on the structure is highlighted in Figure 5 for three different conformations of the water dimer. These three structures have in common the distance of the oxygen atoms (2.98 Å in this example), but they possess different orientations of the hydrogen atoms. The first structure in Figure 5 corresponds to the equilibrium. As can be seen in the figure, the electrostatic interaction energy is strongly influenced by the orientation of the molecules and changes by almost +10 kcal/mol from the hydrogen-bonded structure to the second one that is characterised by parallel dipoles of the two water molecules. Compared to this, the dispersion interaction hardly changes upon a disordering of the hydrogen atoms. While it has a minimum, too, at the equilibrium structure, for the other two structures it lies only +0.7 kcal/mol higher in energy. In line with the structure changes displayed in Figure 4, one can thus conclude that dispersion interactions almost act isotropically and therefore will generally try to maximise the contacts of the interacting sites.

**Figure 6:**
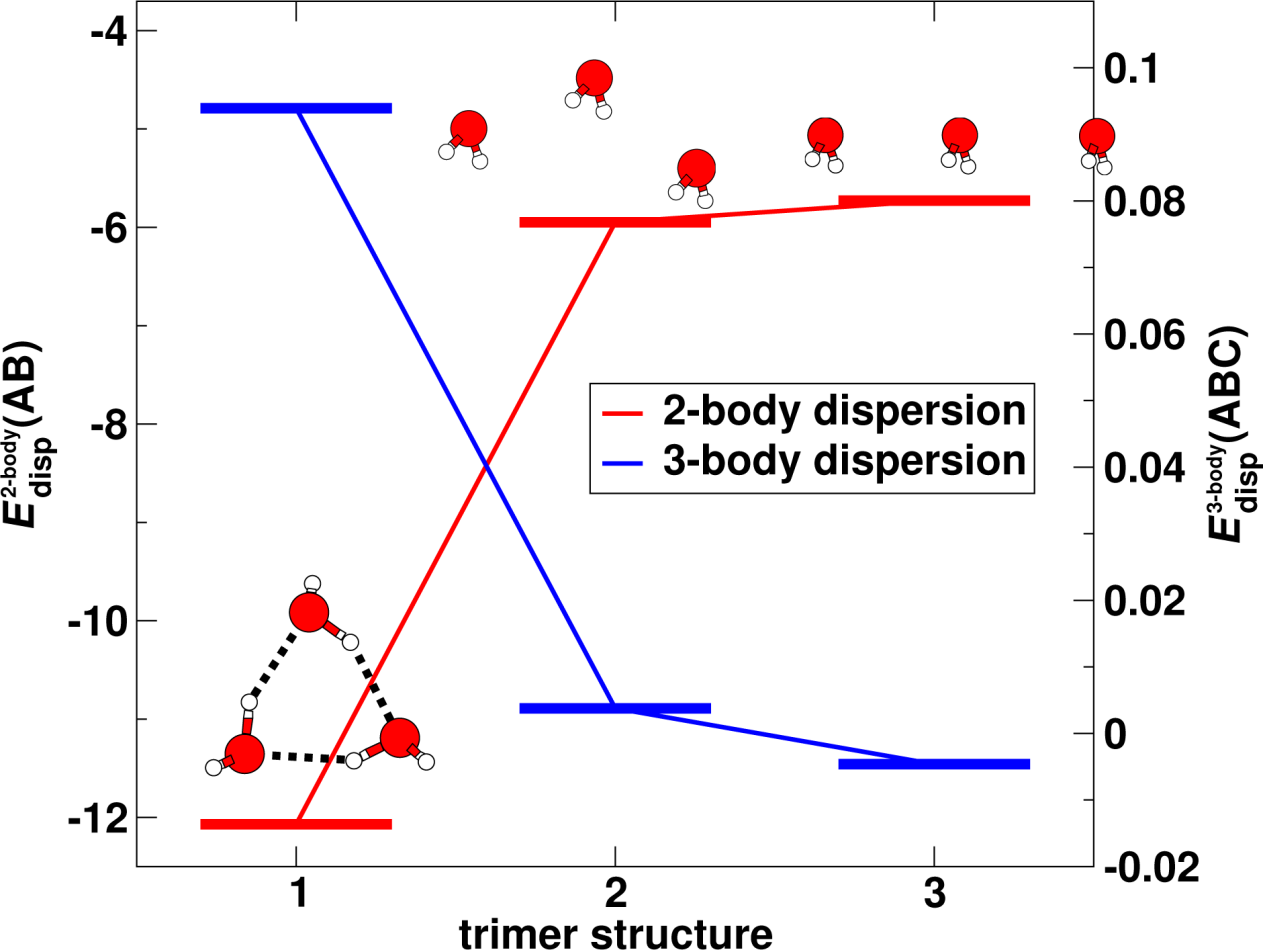
Two- and three-body dispersion energies for three structures of the water trimer. In all conformations the minimum oxygen–oxygen distance amounts to 2.87 Å. The first structure corresponds to the equilibrium. Note that an alternate ordinate scale is used for the two- and three-body energies (aug-cc-pVTZ basis set).

**Figure 7:**
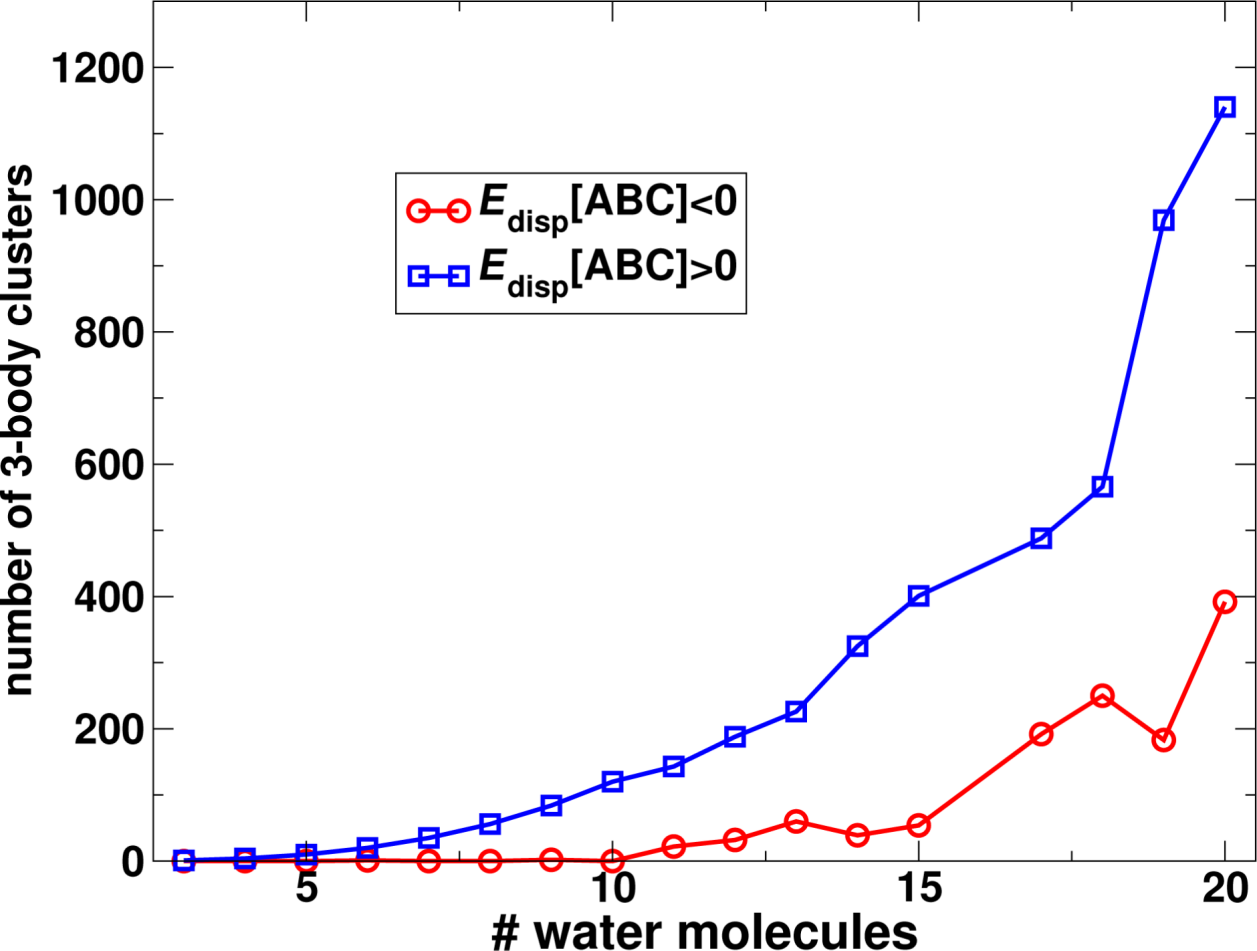
Number of three-body subclusters for which the three-body dispersion energy is attractive (red) or repulsive (blue).

This is also illustrated in Figure 6, which shows the two-body and three-body dispersion energies for three different structures of the water trimer. Here, again structure 1 corresponds to the equilibrium, which is characterised by an equilateral triangle formed by the three oxygen atoms. Compared to this, in structure 2 one of the sides of the triangle is extended, fixing however the other two at a length of 2.87 Å as in the first one. Finally, structure 3 is a linear shaped structure, see Figure 6. As can be seen in the diagram, when the trimer transforms from the most compact equilibrium structure to the linear form, the two-body dispersion interaction strongly reduces by +6 kcal/mol, yet remains attractive having a magnitude of about −6 kcal/mol for the structures 2 and 3. The blue horizontal bars in Figure 6 show the energy levels of the corresponding three-body dispersion energy for the three conformations. As can be seen, it possesses just the opposite dependency on the structure as the two-body dispersion energy. That is, it is repulsive at the triangularly shaped equilibrium structure but turns into an attractive contribution when the structure changes to the linearly shaped form (see the scale on the right-hand side of the diagram in the figure). One can readily describe this anisotropic behavior of the three-body dispersion energy by the simplified Axilrod–Teller form of the interaction energy between three atoms [71]:

[10]
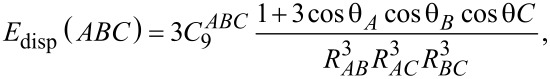


where *R_ij_* and θ*_i_* denote the sides and the angles of the *ABC* triangle and 
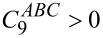
 is a constant coefficient. Equation 10 shows that when *ABC* is in a linear configuration, the three-body dispersion energy is negative (an attractive contribution), while the equilateral triangular configuration leads to a repulsive interaction. Figure 7 shows the number of three-body subclusters that possess a stabilising three-body dispersion energy contribution (determined from the results of the calculations performed in this work). It can be seen that this number grows much less strongly with the cluster size than the number of subclusters with a destabilising three-body dispersion interaction. This qualitatively explains that the total three-body dispersion energy for the respective water clusters considered in this work is always repulsive, too (see below). Furthermore, a comparison of the total magnitudes of the two-body and three-body dispersion energies shows that the three-body dispersion interaction is much weaker than the two-body dispersion interaction. Therefore, its effect on the shape and energies of the larger water clusters can almost be neglected, see also below.

### Many-body interactions in the water trimer and tetramer structures

In this section we analyse the three- and four-body interactions in the cyclic water trimer and tetramer structures, see Figure 2. More precisely, we here want to identify the main interaction energy contributions to the many-body interaction in these two cases. Note, though, that the interaction energy terms of the DFT-SAPT method, see Equation (Equation 5), can only describe the two-body interactions between two subsystems *A* and *B*. While three-body contributions to the DFT-SAPT method have been developed by Podeszwa and Szalewicz [29] many-body effects can, however, be also described by the two-body DFT-SAPT terms with the aid of the pseudodimer technique. For this, recall that the interaction energy of a trimer *ABC* can be approximated by the sum of all mutual two-body interactions:

[11]
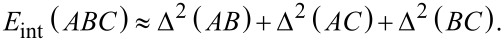


The two-body interaction terms in Equation 11 describe the interactions between two isolated monomers neglecting, however, the perturbation by the third one. Alternatively, two-body interactions can be determined by combining two monomers to one single (pseudo-)monomer and calculating the interaction with the remaining one. In case of the trimer three different possibilities exist:

[12]
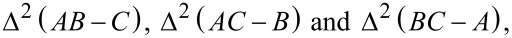


where the term Δ^2^(*AB − C*) now denotes the interaction of the combined system *AB* with monomer *C*. As can be easily understood, the term Δ^2^(*AB* − *C*) contains the two two-body interactions Δ^2^(*AC*) and Δ^2^(*BC*) and a remainder that describes the change of these two interactions due to the perturbation of *A* by *B* and vice versa. This precisely is the contribution that is not described by Equation 11 and, thus, is a contribution to the three-body interaction energy Δ^3^(*ABC*). According to the three different possible pseudodimers, see Equation 12, one can extract the following three terms:

[13]
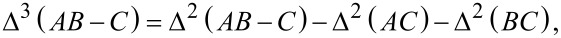


[14]
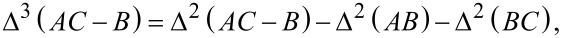


[15]
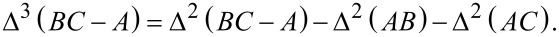


Recall that using the supermolecule method the term Δ^3^(*AB* − *C*) is given by Δ^3^(*AB* − *C*) = *E*(*ABC*) − *E*(*AB*) − *E*(*C*). An insertion of the corresponding total energy expressions into the other terms in Equation 13–Equation 15 shows that

[16]
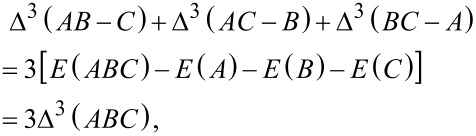


and therefore the sum of the terms Δ^3^(*AB* − *C*), Δ^3^(*AC* − *B*) and Δ^3^(*BC* − *A*) scaled by a factor of one third can be identified as three-body interaction energy.

**Figure 8:**
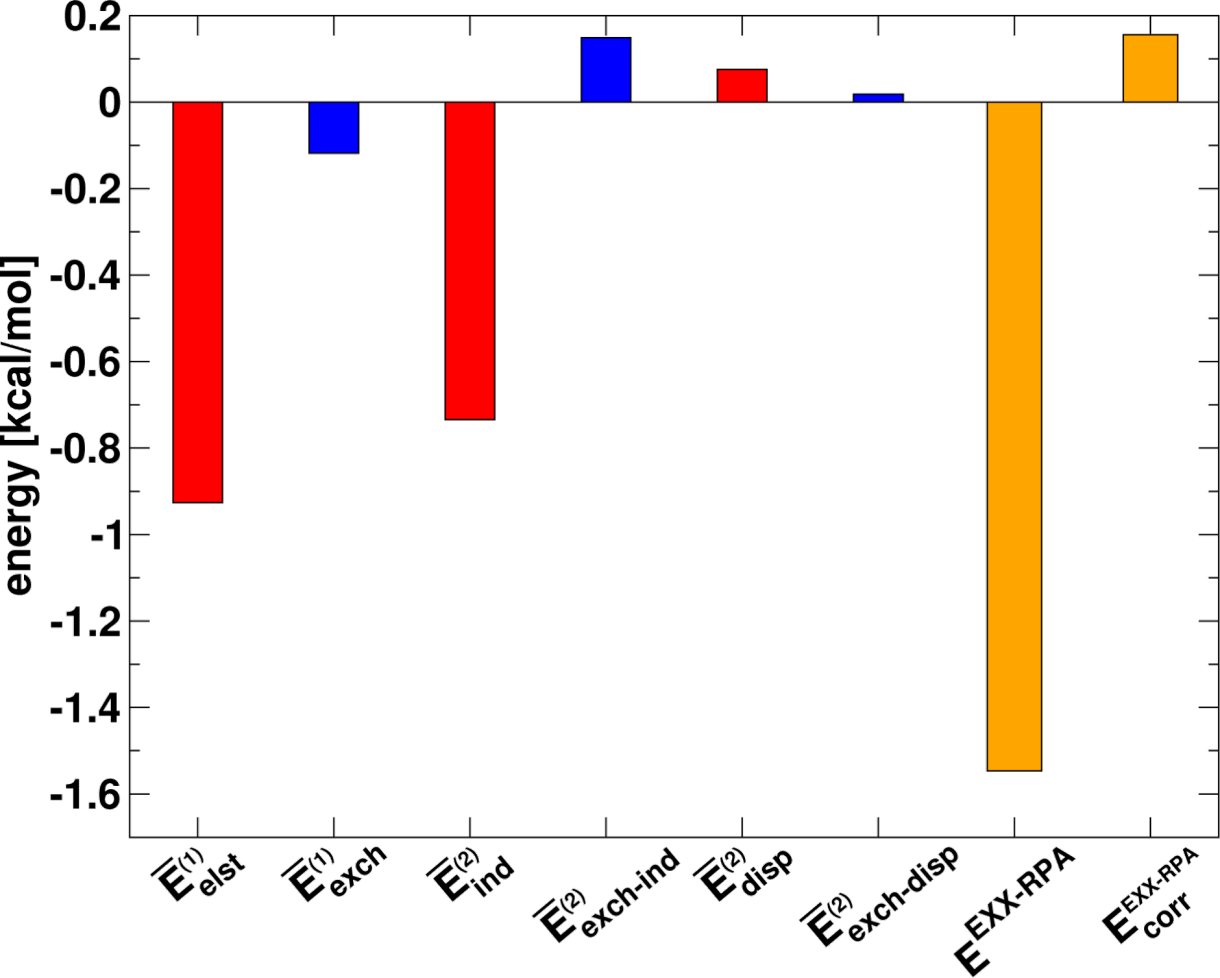
DFT-SAPT energy decomposition of the three-body interaction energy of the water trimer calculated using the pseudo-dimer technique.

We have calculated these terms using the DFT-SAPT method, which allows us to analyse the contribution of the different interaction energy terms of Equation 5 to the three-body interaction energy. The results for the water trimer are shown in Figure 8. In addition, the diagram also contains the three-body interaction energy of the EXX-RPA method as well as its correlation contribution. We have also compared the sum of the terms 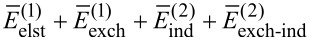 (the overlines are used here to distinguish the terms from the two-body SAPT interaction terms) to the total EXX three-body interaction energy and found a good agreement (DFT-SAPT: −1.63 kcal/mol, EXX: −1.70 kcal/mol). This shows that higher-order interaction contributions to the three-body interaction energy of the EXX method are small.

The diagram in Figure 8 shows that the main contributions to the three-body interaction energy stem from the electrostatic and induction energies. These should not be confused with the standard two-body interaction energy counterparts but should rather be interpreted as the changes of the electrostatic and induction interactions due to the perturbations by another monomer in the trimer. Accordingly, one may interpret the electrostatic contribution in Figure 8 as an induction effect and the induction contribution as a higher-order polarisation effect contained in the three-body interaction energy. The exchange energy counterparts to 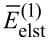 and 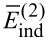 are quite small and strongly cancel each other, making up only a marginal net contribution to the three-body energy. This holds true also for the correlation contribution to the three-body interaction, including the dispersion energy, which is found to be repulsive. Therefore, in line with the findings of the previous section, the many-body correlation effects are negligible in the sum of the different three-body terms, see also Figure 6.

The pseudodimer scheme can also be applied to the tetramer. While this can be done in various ways, in this work we used the following terms:

[17]
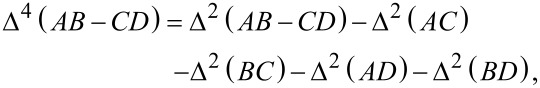


[18]
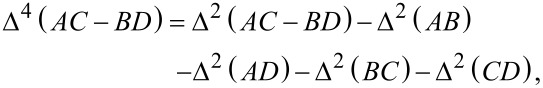


[19]
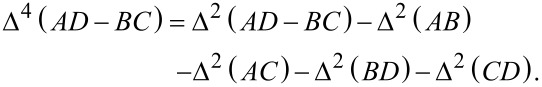


One finds

**Figure 9:**
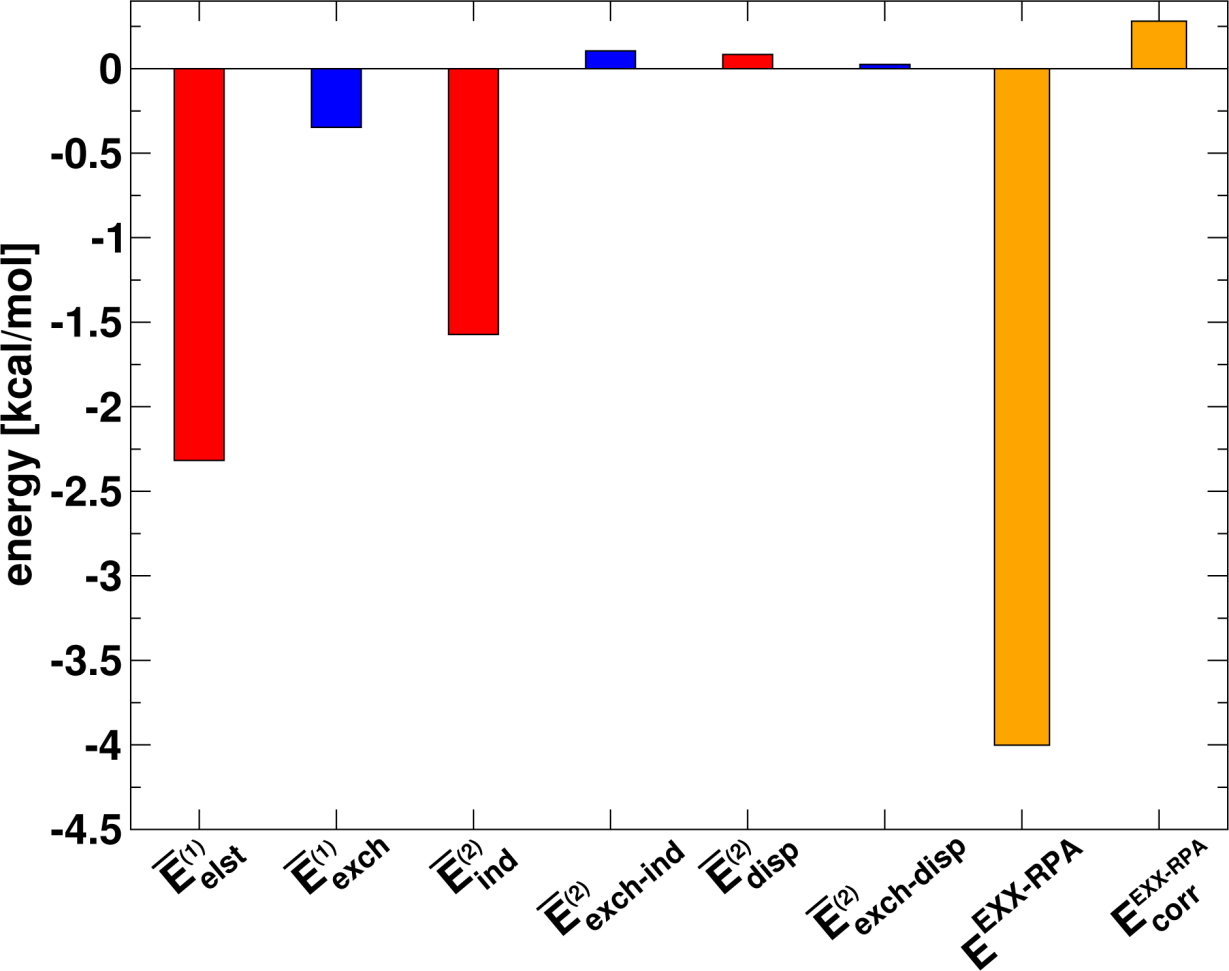
DFT-SAPT energy decomposition of the sum of the three- and four-body interaction energy of the water tetramer calculated using the pseudo-dimer technique.

[20]
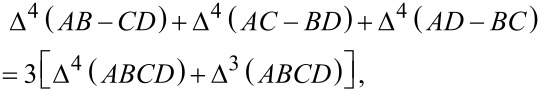


and therefore, analogously to the procedure described above for the trimer, the sum has to be scaled by a factor of one third to reproduce the sum of the three-body and four-body interactions. The DFT-SAPT interaction energy decomposition of the many-body interactions of the water tetramer is shown in Figure 9. While compared to the trimer case the magnitudes of the individual components are distinctly larger, qualitatively the situation is similar to the trimer case. Namely, the main contributions to the many-body interactions in the tetramer stem from the (changes in the) electrostatic and induction energy while again correlation effects are comparably small. The difference between the total EXX many-body interaction energy and the sum of the first and second order energies (excluding the (exchange–)dispersion energy) amounts to −0.15 kcal/mol, which is slightly larger than in case of the trimer. This indicates an increasing importance of higher order interaction energy terms to the many-body interaction energy for larger cluster sizes.

### Dependence of energy contributions on the cluster size

Various contributions to the interaction energy of the water clusters are presented in Table 1 up to the water tridecamer. The second to fourth column show the two-body, three-body and four-body dispersion energies, the fifth column contains the sum of all two-body interactions (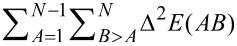) and the seventh column the total (all-body) interaction energy, i.e., *E_N_* = *E*(123…*N*) − 
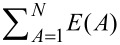
. In addition, the sixth and the eighth column display the correlation contributions to the two-body and all-body interaction energies, respectively.

**Table 1:** Two-, three- and four-body dispersion energies and two-body and total (all-body) interaction energies of the water clusters. All energies are in kcal/mol.

(H_2_O)*_x_*	dispersion			two-body		all-body	
	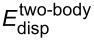	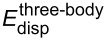	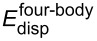	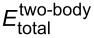	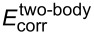	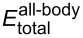	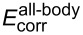

2	−2.28			−4.47	−1.24	−4.47	−1.24
3	−8.02	0.06		−12.22	−4.47	−13.76	−4.31
4	−13.21	0.07	−0.01	−20.27	−6.88	−24.27	−6.61
5	−16.89	0.04	−0.03	−25.62	−8.33	−32.05	−8.17
6 (cage)	−24.09	0.31	−0.04	−35.12	−13.79	−40.35	−12.78
6 (prism)	−24.69	0.33	−0.05	−35.53	−14.25	−40.72	−13.17
6 (ring)	−20.33	0.01	−0.01	−31.63	−10.06	−39.72	−9.60
7	−29.86	0.36	−0.06	−43.64	−17.00	−50.71	−15.74
8	−37.72	0.48	−0.09	−55.15	−21.64	−64.06	−19.99
9	−41.59	0.44	−0.08	−61.60	−23.53	−72.36	−21.72
10	−47.41	0.49	−0.12	−70.27	−27.06	−82.32	−24.82
11	−53.43	0.79	−0.12	−76.72	−31.97	−87.45	−28.86
12	−61.96	0.91	−0.18	−89.79	−37.14	−102.94	−33.33
13	−64.94	0.92	−0.19	−94.26	−38.88	−107.82	−34.93

**Figure 10:**
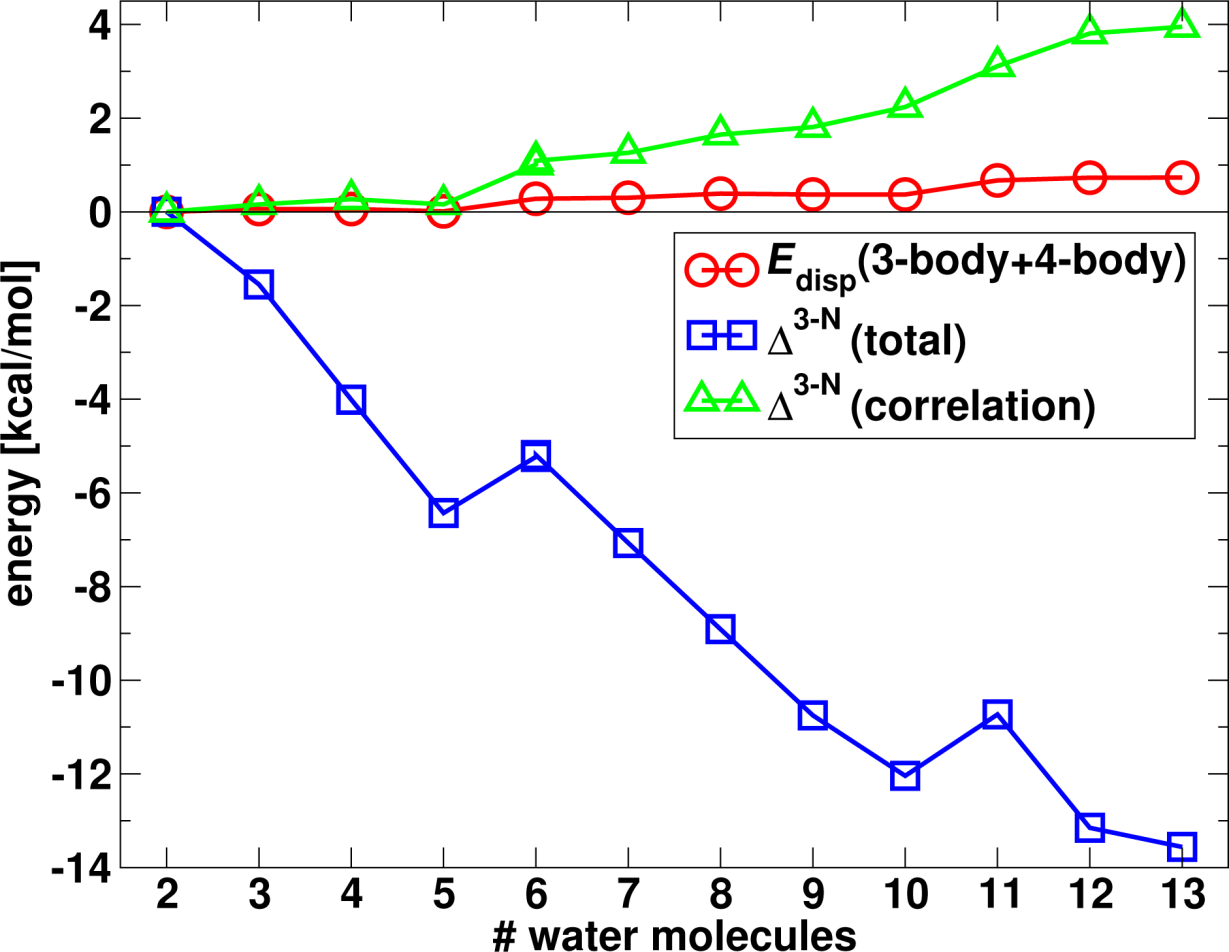
Sum of three- and four-body dispersion interactions compared to the total many-body interactions Δ*^3−N^* in the water clusters.

As can be seen in Table 1, even for large cluster sizes the three- and four-body dispersion energies are fairly small compared to the two-body dispersion interaction. Moreover, since they possess alternate signs, they also partially cancel such that their sum amounts to only 1% of the two-body dispersion at a cluster size of *N* = 13. One can therefore conclude that many-body dispersion effects are negligible for the description of water clusters. This is also illustrated in the diagram in Figure 10 in which the three- and four-body dispersion interaction is plotted along with the total many-body correlation interaction 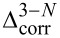, defined by the difference of the 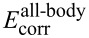 and 
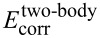
 terms from Table 1. Here one can see that the total many-body correlation interaction, like the many-body dispersion energy, is repulsive and is a significant contribution to the total many-body interaction energy at larger cluster sizes. For instance, for *N* = 13 the correlation contribution reduces the overall attractive many-body interaction energy by 22%.

Yet, how significant are many-body interactions compared to the sum of the two-body interactions in the water clusters? From the results shown in Table 1 one finds that the many-body interactions lead to a lowering of the interaction energy of about 13% for all clusters on average. The only exception to this is found for the cyclic structures of the water tetramer, pentamer and hexamer for which the many-body interaction contributes even 20–25% to the total interaction energy. This strong increase of the many-body interactions in the cyclic structures of water clusters is well known [61]. More recently, Bates et al. [72], Hincapie et al. [73] and Chen et al. [74] have performed high-level coupled-cluster calculations for various isomers of (H_2_O)_6_. These more recent investigations support the findings of earlier studies of the water hexamer [62–63] that the cyclic structures are less favorable than the prism and cage forms in spite of the strong many-body interaction contribution. The results for the three hexamer structures studied in this work indicate why the ring form is less stable than the other two structures. We find a considerably lower stabilisation of the ring-hexamer due to two-body dispersion and (thereby) total two-body interactions by about 4 kcal/mol compared to the cage and prism isomers, see Table 1. This result agrees well with the local molecular orbital energy decomposition analysis of the MP2 interaction energies for the corresponding hexamer structures by Chen and co-workers [74]. Yet, the total interaction energies for the three structures are within a range of 1 kcal/mol (note that compared to this Bates et al. found that the cyclic-chair structure is more unstable by +1.83 kcal/mol than the prism structure using the CCSD(T) method [72]). Thus, the decrease of the two-body interaction energy and the increase of the many-body interaction when switching from the cage/prism form to the ring form of (H_2_O)_6_ almost cancel. Apparently, the water hexamer is the first cluster where the two-body interactions start to dominate the global shape of the cluster geometry, favoring more compact structures than noncompact ring structures.

The above analyis shows that while the many-body interactions in the water clusters are a significant contribution to the total interaction their contribution is scalable and could be modeled, e.g., by scaling the sum of the pair interactions to describe the interactions in the system effectively. This is an approach that is used also in many force field parametrisations for water.

**Figure 11:**
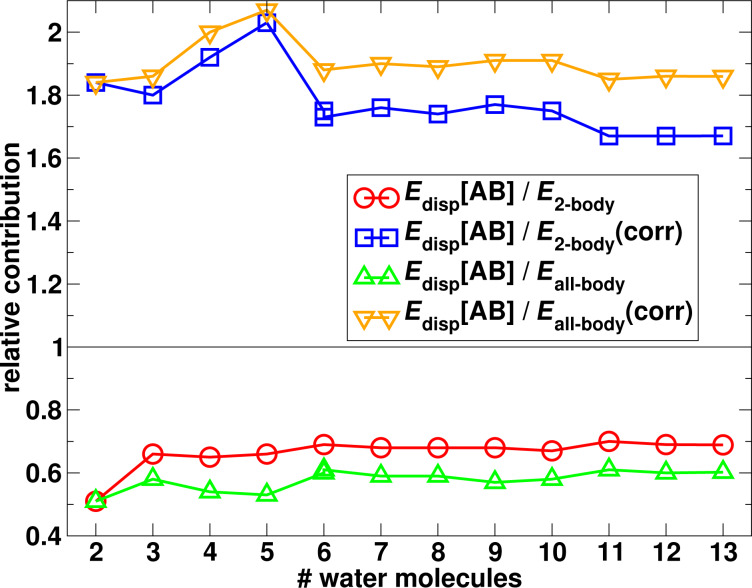
Relative contribution of the two-body dispersion interaction energy to the complete two-body and total (all-body) interaction energies.

In Figure 11 the ratio of the two-body dispersion energy over the two-body and total interaction energies (including the two-body energies) is plotted. One can see that for all cluster sizes the two-body dispersion amounts to about 60% to the total interaction energy and even almost 70% to the total two-body interaction. This clearly demonstrates the significance of dispersion interactions for the stabilisation of the water clusters. The almost constant dependence of the ratio on the cluster size again demonstrates that the increase of the dispersion interaction energy is very similar to the increase of the total interaction energy. Again, this shows that the magnitude of the total interaction energy of the water clusters (in their equilibrium) could be approximated well by a pair-interaction model.

## Conclusion

Different interaction energy contributions to the total interaction energy of water clusters have been analyzed in this work. It has been shown that the main orientation of the water clusters, characterised by the formation of hydrogen bonds, can be well reproduced already on an uncorrelated level using the Hartree–Fock method. However, electron correlation effects to the interaction energy, including the two-body dispersion interaction, lead to a compression of the cluster sizes relative to the structures optimized with the Hartree–Fock method. This global effect originates from the almost isotropic character of the two-body dispersion energy.

Compared to this, the three-body dispersion interaction energy is more strongly dependent on the orientation of the water molecules. However, its was found that dispersion interactions beyond the two-body level are negligible for the description of the stability of water clusters.

The main contributions to the many-body interactions (beyond the two-body level) are described by higher order polarisation interactions, in line with previous studies of the interactions in water clusters [28–29]. This was found through a decomposition of the interaction energies using the DFT-SAPT method with the aid of the pseudodimer technique. Overall, many-body interactions are quite significant and contribute about 13% to the total interaction energy of the water clusters. This amount was found to be almost independent on the cluster size. Because of this, many-body interactions in water should be accurately reproduceable by properly scaled two-body interaction energy terms. Since, however, the many-body polarisation interactions, like their two-body counterparts, may be very anisotropic, this approach needs to be carefully tested also for nonequilibrium structures not considered in this work.

It can generally be concluded that electron correlation effects, including dispersion interactions, are crucial for the description of the two-body interactions in water clusters, yet they yield comparably smaller contributions to the many-body interactions. Efficient computational approaches that are based on the many-body expansion to describe the interactions in water may therefore restrict the description of electron correlation to the two-body level without a severe loss in accuracy.
